# SARS-CoV-2 infection of substantia nigra pars compacta induces expression of miR-330-5p at 10 days post-infection

**DOI:** 10.1099/jgv.0.002149

**Published:** 2025-09-03

**Authors:** Bishwa R. Pokharel, Niska Majumdar, Frank Williams, Abigail Dickerson, Hannah Croy, Jeffrey B. Eells, Alessandro Didonna, Srinivas Sriramula, Paul P. Cook, Shaw M. Akula

**Affiliations:** 1 Department of Microbiology & Immunology, Brody School of Medicine, East Carolina University, Greenville, NC 27834, USA; 2 Department of Molecular Biomedical Sciences, College of Veterinary Medicine, NC State University, Raleigh, NC 27606, USA; 3 Department of Anatomy and Cell Biology, Brody School of Medicine, East Carolina University, Greenville, NC 27834, USA; 4 Department of Pharmacology & Toxicology, Brody School of Medicine, East Carolina University, Greenville, NC 27834, USA; 5 Department of Internal Medicine, Brody School of Medicine, East Carolina University, Greenville, NC 27834, USA

**Keywords:** long COVID, midbrain, miR-330-5p, neuroinflammation, severe acute respiratory syndrome coronavirus 2 (SARS-CoV-2)

## Abstract

Severe acute respiratory syndrome coronavirus 2 (SARS-CoV-2) has been linked to several neurological symptoms in coronavirus disease 2019 (COVID-19) patients; however, the molecular mechanisms underlying virus-induced neuroinflammation are not well identified. For example, the effect of SARS-CoV-2 infection of the substantia nigra pars compacta (SNpc) of the midbrain has not been addressed, in spite of its importance in dopaminergic signalling and neurodegenerative abnormalities. The purpose of this study was to understand the SARS-CoV-2-induced inflammatory response in the SNpc region of the brain. We inoculated (intranasally) transgenic mice expressing human ACE2 under control of the human keratin 18 promoter (K18-hACE-2 mice) with a 4×10^3^ TCID_50_ (mild) dose of SARS-CoV-2. Ten days post-inoculation, SARS-CoV-2 was detected in the SNpc of mice, along with increased levels of IL-1*β*, B1R and ADAM17, and reduced microglial/macrophage occurrence. miR-330-5p expression was significantly reduced in virus-positive SNpc tissue. Luciferase reporter assays supported ADAM17 as a direct target of miR-330-5p. There was no significant difference in miR-330-5p expression levels in the experimental autoimmune encephalomyelitis mice compared to control mice, demonstrating a crucial role for SARS-CoV-2-induced miR-330-5p in brain pathology. Our study uncovers for the first time that SARS-CoV-2 can invade the SNpc and downregulate miR-330-5p expression levels, causing an enhanced ADAM17 expression and possible neuroinflammatory signalling. The results implicate miR-330-5p as a prospective therapeutic target for alleviating midbrain inflammation associated with SARS-CoV-2 infection.

## Introduction

Severe acute respiratory syndrome coronavirus 2 (SARS-CoV-2) causes acute illness called coronavirus disease 2019 (COVID-19) [1]. The COVID-19 pandemic was responsible for millions of deaths worldwide [2]. Patients recovering from COVID-19 have a risk of developing long COVID-19. Long COVID-19 is characterized by neurologic signs and symptoms in humans [3]. Neurologic symptoms may include headache, dizziness, diffuse pain, loss of smell, fatigue, anosmia/ageusia and cognitive dysfunctions [4,5].

 Substantial progress has been made in defining the epidemiology, biology and pathophysiology of long COVID-19 [6,7]. There is a list of biological drivers that may increase the risk of long COVID-19, such as autoreactivity, reactivation of latent viral infections, microvascular dysfunction, systemic inflammation, microbial translocation, metabolic dysfunction and tissue dysfunction [8]. However, it is still elusive as to how such an aberrant impairment of these biological drivers can lead to neurological symptoms. There is evidence that SARS-CoV-2 invades the central nervous system (CNS) [9,11]. We also demonstrated that a low dose of SARS-CoV-2 (4×10^3^ TCID_50_) has the potential to invade both the lung and brain (olfactory bulb) at 10 days post-infection (dPI) of K18-hACE-2 mice as determined by the presence of viral S protein expression by immunostaining experiments [12]. In the same study, it was determined that the lungs and the brain from the above SARS-CoV-2-infected mice were negative for viral S protein expression at 45 dPI. The study concluded that the elevated kinin B1 receptor (B1R) expression may serve as a crux to drive the long-lasting inflammatory response associated with SARS-CoV-2 infection. In addition, we found, in a mouse model, that recovery from SARS-CoV-2 infection could increase the sensitivity of nigrostriatal dopamine neurons to 1-methyl-4-phenyl-1,2,3,6-tetrahydropyridine (MPTP) [13].

 If the presence of SARS-CoV-2 can trigger neuroinflammation, then we hypothesized to observe changes in microRNA (miRNA) levels in the brain at 10 dPI in SARS-CoV-2-infected mice. The goal was to identify miRNAs that may be critical to regulating B1R-induced cell signalling capable of inducing neuroinflammation. MicroRNA is a type of non-coding RNA (~22 bases) capable of regulating more than 60% of all human genes [14]. They typically work at the post-transcriptional level to enhance or repress the translation of mRNA, though some evidence suggests they may have regulatory functions at the level of transcription [15,16]. In this study, we demonstrate that SARS-CoV-2 invades the substantia nigra pars compacta (SNpc) of the K18-hACE-2 mice at 10 dPI and suppresses the expression of miR-330-5p levels critical to supporting a robust ADAM17 expression: a crucial link in the B1R-induced neuroinflammation. The SNpc contains the nigrostriatal dopamine neurons that are critical for normal motor control and, when lost, result in the motor symptoms of Parkinson’s disease [17]. Overall, our results from using *in vivo* and *in vitro* models clarify a crucial role for SARS-CoV-2-induced decrease in miR-330-5p and a corresponding increase in ADAM17 expression levels in the SNpc to serve as a milieu capable of supporting virus-induced neuroinflammation.

## Methods

### Virus

All the work pertaining to the use of SARS-CoV-2 was performed in a BSL-3 laboratory. SARS-CoV-2 (isolate USA-WA1/2020; BEI Resources, Manassas, VA, USA) was propagated, purified and titrated in Vero cells as per the standard protocols [18]. The following research was approved by the Office of Prospective Health/Biological Safety for the use of biohazardous agent (SARS-CoV-2) and the registration number is 20–01 (title: Host response to COVID-19 infection in Eastern North Carolina).

### Infection assays

The virus concentration in the mouse midbrain specimens was detected by reverse transcriptase quantitative PCR using the 2019-nCoV RUO Kit as per the protocols outlined by the manufacturer (Integrated DNA Technologies, Coralville, IA). Briefly, RNA from the midbrain specimens was extracted using QIAmp viral RNA kits (QIAGEN, Georgetown, MD). The viral copy numbers in the extracted RNA were determined by reverse transcriptase qualitative PCR using the 2019-nCoV RUO Kit as per earlier protocols [19].

### Cells

Human embryonic kidney cells (HEK-293 T) were propagated in Dulbecco Modified Eagle Medium (Invitrogen, Carlsbad, CA) containing 10% charcoal-stripped FBS, l-glutamine and antibiotics as per earlier standard protocols [18].

### Animal study design

We used transgenic mice with the human ACE2 gene under the control of the K18 promoter (B6.Cg-Tg(K18-ACE2)2Prlmn/J, Jackson Labs, Strain #034860) in this study. This animal model has been previously characterized and shown to recapitulate key features of SARS-CoV-2 pathogenesis, including high viral replication in the lungs and, in some cases, neuroinvasion and CNS inflammation [12,20, 21]. Male and female K18-hACE2 mice, ranging in age from 4 to 6 months, were randomly assigned to receive either vehicle saline (mock controls) or SARS-CoV-2 at a dose of 4×10^3^ TCID_50_. This is considered a low dose of SARS-CoV-2. We have successfully used 4×10³ TCID_₅0_ as a low infectious dose in the context of SARS-CoV-2 infection of the K18-hACE-2 transgenic mouse model [12]. We determined that 80% of the mice that were infected with a 4×10³ TCID₅₀ dose survived. This model has been used successfully by us to study long-lasting effects of COVID-19 [22]. Mice were anesthetized with 3–4% isoflurane, and 12.5 ml of either the vehicle or SARS-CoV-2 was pipetted into each naris (25 µl). Following viral inoculation, mice were housed individually and managed in an ABSL3 facility (East Carolina University, Greenville, NC). Mice were weighed daily and monitored for symptoms. On the tenth day post-inoculation, they were euthanized, and tissues were harvested.

 Experimental autoimmune encephalomyelitis (EAE) was induced in C57BL/6 female mice of 8–10 weeks of age with 100 µg myelin oligodendrocyte glycoprotein 35–55 peptide in complete Freund’s adjuvant as described before [23]. Control mice received everything but the peptide.

### Tissue dissection

For isolation of the SNpc, the frozen midbrain was mounted on a custom tissue slicer. Frozen sections, ~600-µm-thick frozen section starting around 4 mm from bregma, were mounted on a glass slide and then a 1-mm biopsy punch was used to collect the SNpc as previously described [24]. RNA was isolated using the ExoBrite™ EV Total RNA Isolation Kit from Biotium according to the manufacturer’s protocol.

### Reverse transcriptase quantitative PCR

Reverse transcriptase quantitative PCR was performed as per earlier studies [18]. The specific forward primer to amplify miR-330-5p was 5′-TCTCTGGGCCTGTGTCTTAGGCAAAA-3′. The forward primer to amplify miR-93-5p was 5′-CAAAGTGCTGTTCGTGCAGGTAGAA-3′. The forward and reverse primers used to amplify different target genes of miRNAs are provided in Table S1, available in the online Supplementary Material. Data were excluded from further analyses if CT values exceeded 35 and/or the amplification score was below 1.5.

### Immunohistochemical staining

Mouse midbrains were fixed in 10% normal buffered formalin, then dehydrated through graded ethanol, defatted in xylene and embedded in the coronal plane in paraffin (Paraplast‐Xtra; Fisher Scientific, Waltham, MA, USA). 10‐μm sections (five sections per slide) were cut and mounted onto Superfrost‐Plus slides (Fisher Scientific). The sections on these slides were immunostained for tyrosine hydroxylase (TH) (1 : 250; mouse monoclonal, T1299; Sigma‐Aldrich, St. Louis, MO, USA) and ionized calcium‐binding adapter molecule 1 (IBA1), which labels microglia and macrophages [25] (1 : 200 dilution; rabbit polyclonal, 019‐19741; Wako, Richmond, VA, USA) using immunofluorescence [12] and polyclonal anti-SARS Coronavirus (anti-serum, Guinea Pig; NR-10361; obtained from BEI resources, Manassas, VA, USA; 1:500 dilution) protocol as per our early studies [22]. The staining was conducted to determine the relative changes in the levels of microglia/macrophages in SARS-CoV-2-infected mouse midbrain compared to uninfected. A qualitative assessment of immunofluorescence, across 4–6 sections, was used to compare TH expression in control and SARS-CoV-2-infected sections. Fiji ImageJ was used to compare the distribution of IBA1 expression across sections across equal areas of tissue.

### Luciferase reporter assay

The oligos corresponding to the 3′-UTR (with WT binding sites for miR-330-5p) of ADAM17 (accession number: NM_009615.6) and FGF7 (accession number: NM_008008.4) were synthesized and cloned into the XhoI/XbaI site located at 3′-UTR of pmirGLO dual-luciferase vector (Promega, Madison, WI, USA). HEK-293T cells were plated onto 6-well plates. At 24 h post-plating, HEK-293T cells were co-transfected with the above-mentioned reporter plasmids alone or in the presence of respective miRNA mimics or scrambled controls (NC) using FuGene HD (Promega). At 48 h post-transfection, the Renilla luciferase activity was measured using the dual luciferase reporter assay system (Promega) as per the manufacturer’s recommendations. The oligos tested in this study are provided in Table S2.

## Results

### Low dose of SARS-CoV-2 can invade SNpc of K18-hACE-2 mice

**Fig. 1.**
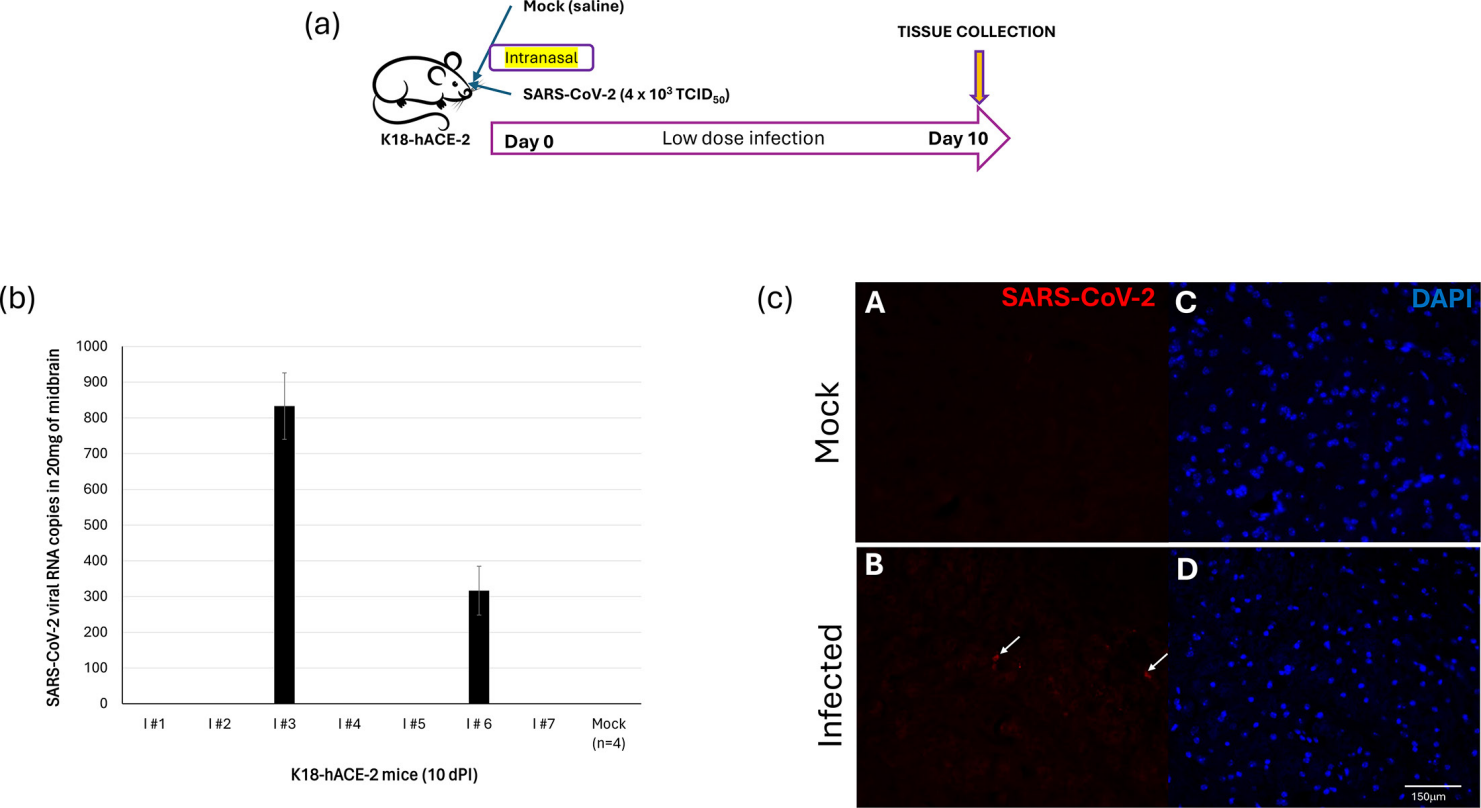
SARS-CoV-2-inoculated K18-hACE-2 SNpc is positive for viral RNA. (**a**) A schematic depicting an animal study design. (**b**) Monitoring SARS-CoV-2 litres in the SNpc of virus-inoculated K18-hACE-2 mice on 10 days post-inoculation by reverse transcriptase quantitative PCR. RNA was extracted from the SNpc of mock and SARS-CoV-2-infected mice. RNA concentrations were measured with a NanoDrop ND-2000 spectrophotometer (Thermo Fisher Scientific, Waltham, MA, USA). The virus concentration in the specimens was detected by quantitative PCR monitoring nucleocapsid *N* gene using the SARS-CoV-2 (2019-nCoV) CDC quantitative PCR Probe Assay (Integrated DNA Technologies). The limit of detection for this assay is 50 copies. (**c**) A representational immunofluorescence image of infected mice (I#3) SNpc positive for S protein of SARS-CoV-2 (arrowheads) compared to SNpcs derived from mock-inoculated mice.

There are several studies to demonstrate that SARS-CoV-2 can infect forebrain and hindbrain regions of mice [26,28]. There is limited research on the ability of SARS-CoV-2 to infect the midbrain region of mice. In this study, we inoculated K18-hACE-2 mice with a low dose of SARS-CoV-2 or mock (saline)-inoculated intranasally (Fig. 1a). We observed an 80% survival rate in mice following inoculation with a low dose of SARS-CoV-2 compared to mock-inoculated mice. We did not observe any significant body weight loss in these inoculated animals. Interestingly, only two out of seven mice (mice ID: I#3 and I#6) that were inoculated with a low dose of SARS-CoV-2 were positive for the virus in their SNpc by 10 dPI. The presence of SARS-CoV-2 in the SNpc was confirmed by PCR (Fig. 1b). In addition, the expression of the spike (S) protein of SARS-CoV-2 was detected in SNpc (derived from mice I#3 and I#6) when immunostained with antibody specific to SARS-CoV-2 encoded S protein compared to specimens derived from mock-inoculated (Fig. 1c). These data demonstrate that mice expressing hACE2 are susceptible to intranasal infection of SARS-CoV-2 virus and that there can be variation in the ability of SARS-CoV-2 to invade the CNS.

### SARS-CoV-2 infection of SNpc-induced inflammation

**Fig. 2.**
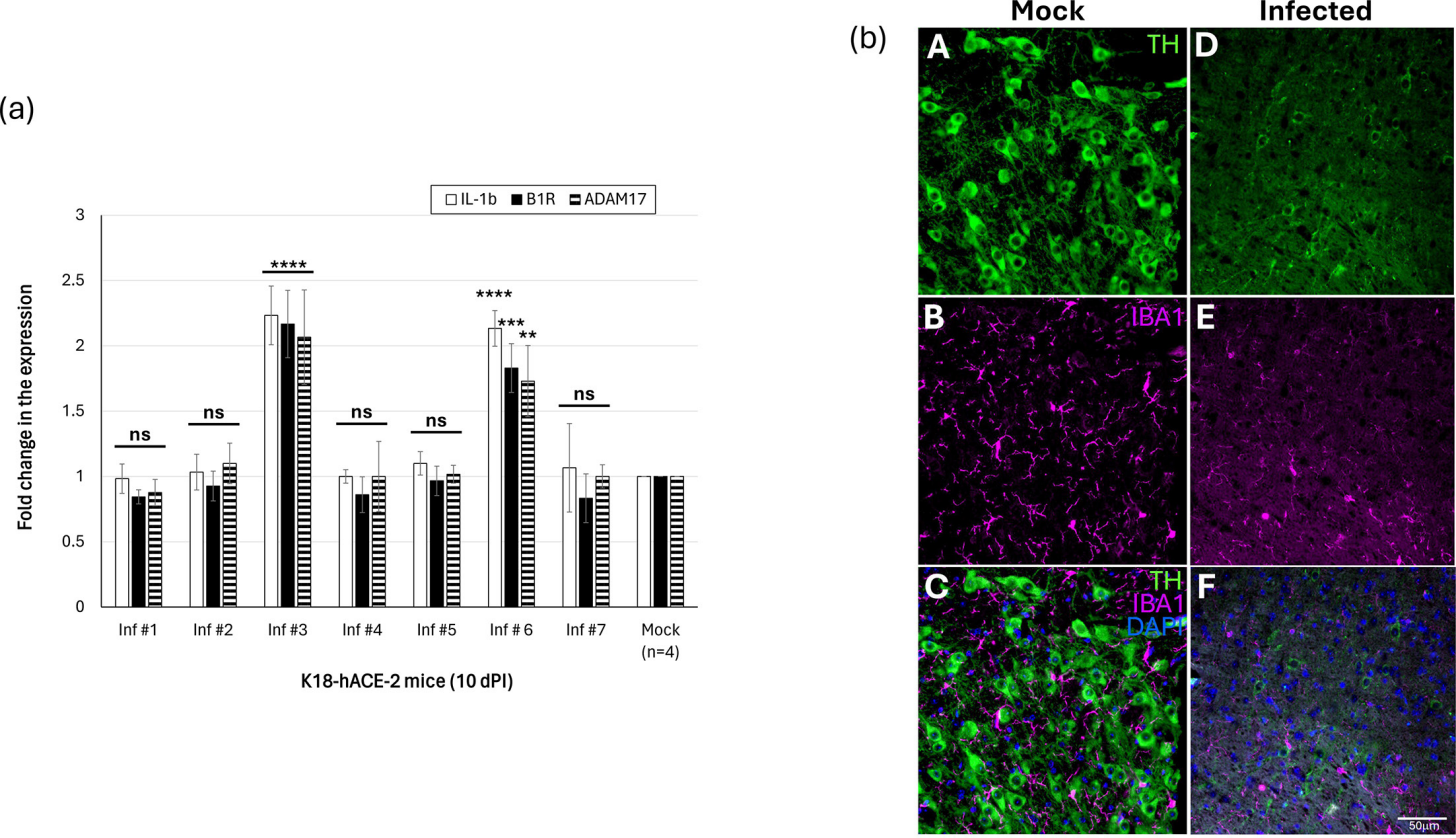
SARS-CoV-2 invasion of the brain induces inflammation. (**a**) Reverse transcriptase quantitative PCR was performed using RNA extracted from the midbrain sections of SARS-CoV-2-inoculated K18-hACE-2 mice on 10 days post-inoculation. We monitored expressions of IL-1*β*, B1R and ADAM17 using specific primers. Data are the mean±sd (error bars) of three experiments. ANOVA was performed using IBM SPSS v26 (Cary, NC) to determine significant differences between the different treatment and control groups, followed by the Tukey HSD post hoc test for multiple comparisons. ‘****’, ‘***’, ‘**’ and ‘ns’ denote *P*<0.0001, *P*=0.0002, *P*=0.0059 and not significant, respectively. (**b**) Expression levels of dopamine neurons (TH) and microglia (IBA1) were significantly lowered in SARS-CoV-2-infected SNpc sections compared to mock controls. A representational figure from mouse I#3 is shown.

Host response to any form of viral infection results in inflammation. The expression levels of known inflammatory markers, B1R and IL-1*β*, were elevated in only two (out of the seven mice, Fig. 1b) of the virus-inoculated K18-hACE-2 mice that were positive for SARS-CoV-2 in SNpc (I#3 and I#6, Fig. 2a). The downstream target of B1R, ADAM17, was also observed to be elevated in two mice that were positive for SARS-CoV-2 in their brain (Fig. 2a). There is an inverse correlation between inflammation and microglia/macrophage activation [29]. Therefore, we performed immunohistochemical staining of the paraffin-embedded SNpc sections of the mice to determine the expressions of TH and microglia/macrophages (IBA1). A mild SARS-CoV-2 infection of K18-hACE-2 midbrain resulted in a qualitative reduction in whole body microglia/macrophages (soma+branches) in SNpc (Fig. 2b). This observation is based on descriptive immunostaining and was not quantitatively measured. Therefore, further studies using quantitative analyses are needed to validate and quantify these changes. A representational low magnification image showing the distribution of TH immunoreactive neurons in the SNpc is shown in Fig. S1. Microglia/macrophages serve as the principal immune cells within the CNS, crucially contributing to neuronal health and synaptic remodelling [30]. Dysregulation or diminishment of microglia/macrophage surveillance capacity may lead to compromised immune response and increased susceptibility to neuroinflammation [31].

### miR-330-5p levels were decreased in SARS-CoV-2-infected brains in K18-hACE-2 mice

**Fig. 3.**
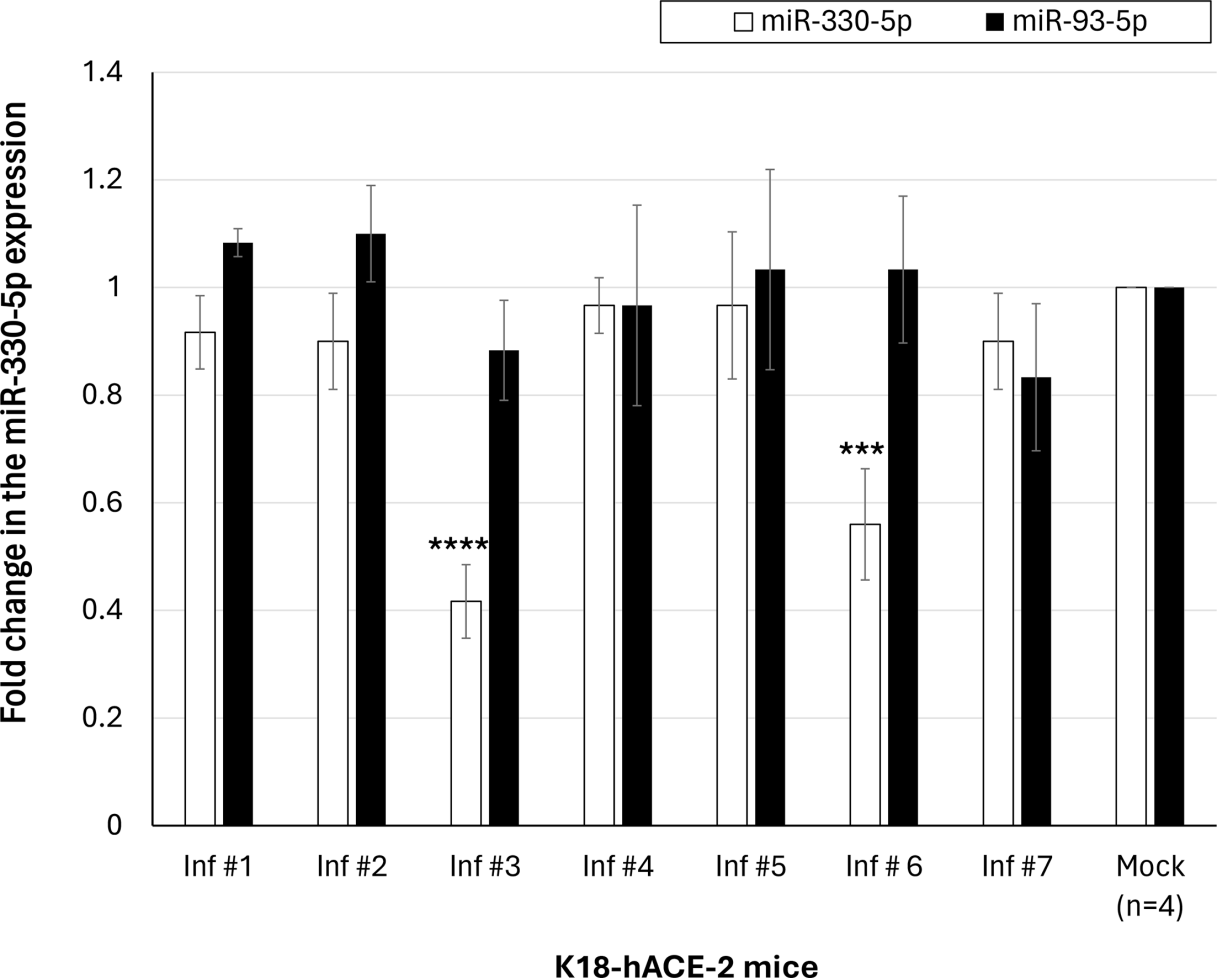
miR-330-5p levels were significantly lowered in SARS-CoV-2-infected brains in a subset (2 of 7) of infected K18-hACE-2 mice. The fold change in the miR-330-5p expression in SARS-CoV-2-infected mice is relative to the average expression of the respective miRNA in mock-inoculated mice, and that is considered to be 1-fold. Expression of miR-93-5p was used as a control. The average±sd of five individual experiments is listed above as the data points. ANOVA was performed using IBM SPSS v.26 (Cary, NC) to determine significant differences between the different treatment and control groups, followed by the Tukey HSD post hoc test for multiple comparisons. ‘****’ and ‘***’ denote *P*<0.0001 and *P*=0.0002, respectively.

The idea to specifically examine the levels of miR-330-5p in the SNpc region of SARS-CoV-2-infected mice was due to two main reasons: (i) our screening experiment performed using next-generation sequencing on RNA derived from SARS-CoV-2-infected mice brains. The miR-330-5p levels in the brain of SARS-CoV-2-infected mice were significantly lower than in mock-inoculated mice (Fig. S2); (ii) the 3′-UTR of ADAM17 contained a binding site for miR-330-5p (Fig. S3). Therefore, we monitored the expression of miR-330-5p in the SNpc regions of SARS-CoV-2-inoculated mice. miR-330-5p levels were significantly lower in SARS-CoV-2-infected SNpc compared to those brains that were negative for SARS-CoV-2 and mock-inoculated (Fig. 3). In other words, among the seven SARS-CoV-2-inoculated mice, only two animals that were positive for SARS-CoV-2 in SNpc (I#3 and I#6) showed robust downregulation of miR-330-5p. The remaining animals did not exhibit significant differences from mock controls at 10 dPI. Also, the expression of miR-93-5p (used as a negative control) was not significantly altered in the brains of SARS-CoV-2-infected mice. We observed an inverse correlation between miR-330-5p and ADAM17 expression levels. EAE is a commonly used animal model for studying multiple sclerosis. Interestingly, expression of miR-330-5p was not significantly altered in the EAE mice brain compared to control mice (Fig. S4).

### miR-330-5p is a potential regulator of ADAM17 expression

**Fig. 4.**
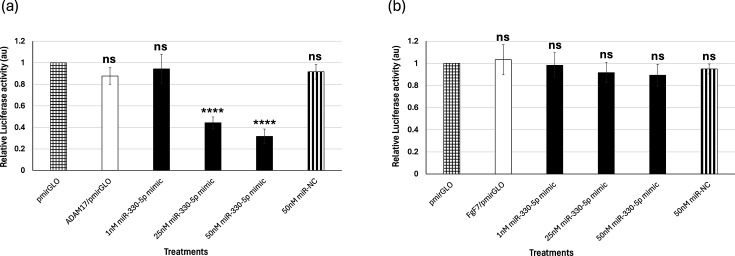
miR-330-5p directly interacts with its target, ADAM17 mRNA. A dual luciferase reporter assay was performed to demonstrate the interactions between miR-330-5p and its putative targets, ADAM17 (**a**) and FGF7 (**b**) mRNA (control) in HEK-293T cells. The *x*-axis indicates the different treatments, while the *y*-axis denotes the relative luciferase activity. Bars represent average±sd of three individual experiments. ANOVA was performed using IBM SPSS v.26 (Cary, NC) to determine significant differences between the different treatment and control groups, followed by the Tukey HSD post hoc test for multiple comparisons. ‘****’ and ‘ns’ denote *P*<0.0001 and not significant, respectively.

Luciferase reporter assay was performed to confirm the ability of miR-330-5p to interact with its putative targets. The reporter assay demonstrated the ability of miR-330-5p to physically interact with the 3′-UTR of ADAM17 (Fig. 4a). miR-330-5p mimic, when used at concentrations of 25 nM and 50 nM, specifically inhibited luciferase activity compared to 50 nM of miR-NC. Also, miR-330-5p mimic could not alter luciferase activity when the 3′-UTR of FGF7 (irrelevant target for miR-330-5p and used as a control) was used as the target sequence, further demonstrating the specificity of the miR-330-5p interactions (Fig. 4b).

## Discussion

SARS-CoV-2 was originally identified as a virus that affects the respiratory system, with less evidence of its impact on the CNS [32]. The capacity of SARS-CoV-2 to infect the nervous system has been a subject of persistent debate, especially considering multiple reports indicating neurological symptoms in patients [33,34]. During the early stages of the pandemic, a unique instance occurred in which a patient manifested meningoencephalitis without exhibiting respiratory distress symptoms [35], underscoring the vulnerability of the CNS to this virus.

To date, comprehensive research has primarily focused on the forebrain and hindbrain, studying the presence and effects of SARS-CoV-2 infection [36,37]. These investigations determined that SARS-CoV-2 has affinity for neural cells, causing neuroinflammation, neuronal apoptosis and disruptions in neurotransmission pathways [38]. Thorough research of the forebrain and hindbrain has provided important insights into the virus-induced neurological effects; however, the midbrain remains relatively underexplored, resulting in a major gap in understanding SARS-CoV-2’s overall effect on the CNS, specifically given the midbrain’s central role in neurological and behavioural functions.

To address this gap in our understanding of SARS-CoV-2 biology, our laboratory has performed research using a validated mouse model infected with a low dose of SARS-CoV-2. A previous study conducted by our group using the same mouse model (K18-hACE-2) showed the presence of SARS-CoV-2 in the olfactory lobe, thereby confirming the model’s suitability for CNS infections [12]. In this report, we employed infection assays, cloning, luciferase reporter assays, imaging and reverse transcriptase quantitative PCR techniques to demonstrate SARS-CoV-2 successfully invading the midbrain of the mice (Fig. 1).

The presence of SARS-CoV-2 in the SNpc resulted in inflammation as observed by a substantial increase in the expression levels of proinflammatory genes, IL-1*β*, B1R and ADAM17 (Fig. 2). A key observation in our study is that only two out of seven inoculated animals (less than 50%) demonstrated detectable levels of SARS-CoV-2 RNA and associated neuroinflammatory changes in the midbrain at 10 dPI (Figs1  2). Interestingly, we performed a group-wise analysis by combining data from all seven inoculated animals (Figs2  3) and comparing them to the mock group (*n*=4). When all inoculated animals were averaged and compared to mock controls, there was no statistical significance (*P*>0.05) observed (*data not shown*). This is primarily due to the high inter-animal variability within the inoculated group – specifically, the strong neuroinflammatory response observed only in two out of the seven SARS-CoV-2-infected animals. Our results align with findings from other studies that demonstrated heterogeneous CNS involvement during SARS-CoV-2 infection [39,40]. Based on the data, we concluded that the K18-hACE-2 model exhibits biological variability in CNS involvement, which mirrors observations in humans, where only a subset of SARS-CoV-2-infected patients develops neurological symptoms or detectable neuroinflammation.

The equivalent RNA copy numbers measured by PCR of the input titre of TCID_50_ in the inoculum were 2×10^4^. In other words, our purified SARS-CoV-2 comprised 80% of non-infectious virus particles. This is expected in virus replication because of the following reasons: (i) virus replication is an error-prone process primarily due to the lack of proofreading activity [41]; (ii) it is reported that greater than 90% of virus particles produced during viral replication are non-infectious [42,43]; (iii) defective viral genomes are prevalent in RNA viruses like SARS-CoV-2 and are considered replicative defective and non-infectious [44,45]. However, it will be difficult to extrapolate this ratio with the RNA copy numbers reported in Fig. 1b because (i) the non-infectious particles would have failed to infect mice, (ii) the viral RNA copy numbers are low and (iii) it is hard to determine as to what percentage of active viral replication has occurred in the brain since neuroinvasion.

This study is the first to report a direct regulatory connection between miR-330-5p and ADAM 17 in relation to midbrain neuroinflammation caused by SARS-CoV-2 infection. Our results reveal an inverse correlation between the expression of miR-330-5p and ADAM 17, showing that the lower expression of miR-330-5p is directly correlated with higher expression of ADAM 17 during SARS-CoV-2 infection in the midbrain (Fig. 3). Viral pathogens can trigger differential expressions of several host miRNAs to evade inflammation [46,47]. Our luciferase reporter assay and reverse transcriptase quantitative PCR results validate the binding affinity and functional regulation of miR-330-5p to its target, the 3′-UTR of ADAM 17 (Fig. 4). These results reveal a novel regulatory mechanism that could alter neuroinflammation in the midbrain during SARS-CoV-2 infection.

Our earlier study recognized a pathway linking the upregulation of B1R, which directly influences ADAM17 expression during neuroinflammation due to SARS-CoV-2 in the same mouse model [12]. Our current findings implicate miR-330-5p as a central regulatory biomolecule affecting ADAM 17 expression precisely in the SNpc. Since the loss of dopamine neurons in the SNpc causes the motor symptoms of Parkinson’s disease and early evidence suggests that SARS-CoV-2 infection may be a risk factor for Parkinson’s disease, dysregulation of ADAM 17 could be a mechanism through which SARS-CoV-2 infection induces inflammation to damage dopamine neurons.

We acknowledge two specific limitations of this study. First, it is to do with the use of the correct mouse model to study SARS-CoV-2 infection of the brain. For example, the Syrian hamster model offers better representation of respiratory pathology, including lung injury and spontaneous recovery, closely mimicking moderate human disease [48,49], but is limited in replicating long-term neurological outcomes or CNS viral dissemination [50]. On the contrary, the K18-hACE-2 mouse model is a powerful tool for studying SARS-CoV-2 neuroinvasion, but it does not fully represent the complexity of human COVID-19. The transgenic overexpression of hACE2 under the K18 promoter results in high viral susceptibility in neuronal tissues, potentially exaggerating CNS involvement relative to typical human infections [20,28]. Therefore, our findings should be interpreted in the context of the K18-hACE-2 model-specific constraints. Second, the specific limitations of this study primarily are regarding the duration, low dose and degree of SARS-CoV-2 exposure. Even though our low-dose exposed mouse model successfully demonstrates midbrain infection and provides early understandings into miRNA and gene interactions, prolonged-duration and higher-dose infection of SARS-CoV-2 research would be helpful for an in-depth understanding of long exposure effects of the virus and potential adaptive responses within gene regulatory systems.

Future studies will be directed towards understanding (i) the miR-330-5p and ADAM17 expression levels in response to SARS-CoV-2 infection of other regions of the brain and organs like the lungs. Such studies will help us to better define their (miR-330-5p/ADAM17-induced signalling) role in systemic vs. localized responses to infection; (ii) the selective viral invasion of the midbrain reported in our study as SARS-CoV-2 could only invade midbrain of only two out of seven mice; and (iii) the effect on astrocyte function, vascular damage and disruption of the blood–brain barrier. Examining the causes of this selectivity in viral infection might help us elucidate host susceptibility affecting viral tropism and sensitivity in the midbrain. This favoured infection pattern parallels human infection, best shown by the individual adaptations in long COVID-19 development. We propose miR-330-5p to serve as a potential therapeutic target for reducing SARS-CoV-2-associated neurological complications.

## Supplementary material

10.1099/jgv.0.002149 Uncited Supplementary Material 1.

## Abbreviations

B1R: kinin B1 receptor
CNS: central nervous system
dPI: days post-infection
EAE: experimental autoimmune encephalomyelitis
IBA1: ionized calcium‐binding adapter molecule 1
miRNA: microRNA
NC: scrambled controls
S: spike protein
SARS-CoV-2: severe acute respiratory syndrome coronavirus 2
SNpc: substantia nigra pars compacta
TH: tyrosine hydroxylase
