# Supplementary material for: SARS-CoV-2 infection of substantia nigra pars compacta induces expression of miR-330-5p at 10 days post-infection
Source: J Gen Virol. 2025 Sep 3;106(9):002149. doi: 10.1099/jgv.0.002149 (PMC12451610; doi:10.1099/jgv.0.002149)
Supplement: Uncited Supplementary Material 1. [file jgv-106-02149-s001.pdf]

Supplementary Table 1: Primers used for RT-qPCR

**Target gene**	**Description**	**Primer**	**Tm**
ADAM17	Forward	5’ - CCATAGCTGTGAGTGGCGAT - 3’	59.9˚C
	Reverse	5’ - TCCTTCATCCACCCTCGAGT - 3’	60˚C
B1R	Forward	5’ – TTCCGTCAAAGTCGTCCCTG - 3’	60˚C
	Reverse	5’ – CCAATGGGAGGAGGAAACCC - 3’	60˚C
GAPDH	Forward	5’ – TGTAGGCCAGGTGATGCAAG - 3’	60˚C
	Reverse	5’ – TACGCATTATGCCCGAGGAC - 3’	60˚C

Supplementary Table 2. Oligos Used to Construct Luciferase Reporter Vectors.

Target Gene	Oligos
ADAM17	**ADAM17.F**<br>5'-AAACTAGCGGCCGCTAGTACTCTGTAACTCCAATCCCAGAGGAGATT- 3'<br><br>**ADAM17.R**<br>5' -CTAGAATCTCCTCTGGGATTGGAGTTACAGAGTACTAGCGGCCGCTAGTTT- 3'
Fgf7	**FGF7.F**<br>5' -AAACTAGCGGCCGCTAGTAAGACTGCATTAAAGAAAGATTTGAAGAAT- 3'<br><br>**FGF7.R**<br>5' -CTAGATTCTTCAAATCTTTCTTCCTGCAGTCTTACTAGCGGCCGCTAGTTT- 3'

* miR-330-5p binding site is highlighted in RED (underlined).

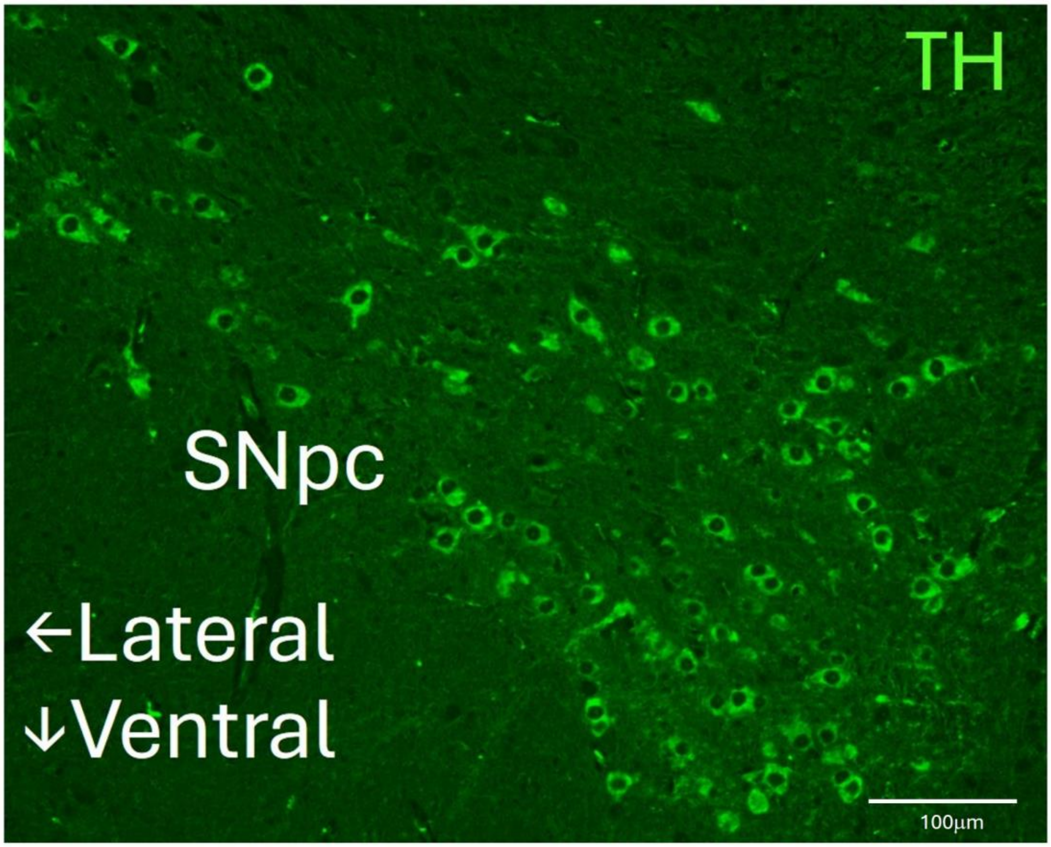

Supplementary Figure 1. A low magnification image shows the distribution of tyrosine hydroxylase (TH) immunoreactive neurons in the substantia nigra pars compacta (SNpc) and demonstrates the approximate rostral-caudal level of high magnification images taken in **Figure 2**.

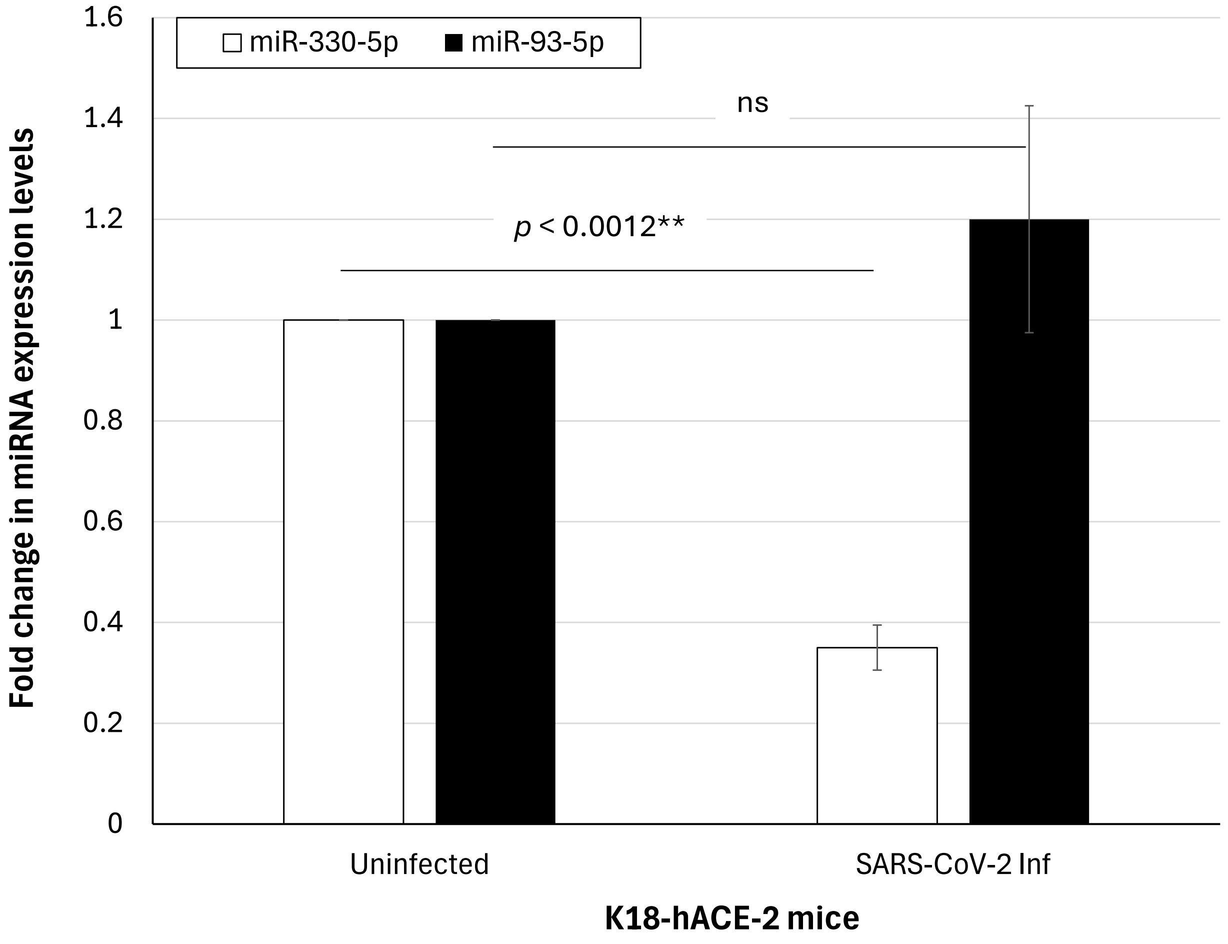

Supplementary Figure 2. NGS determined the fold change in the miR-330-5p expression in SARS-CoV-2-infected mice (*n = 5*) compared to uninfected mice (*n = 5*) and that is considered to be 1-fold. Expression of miR-93-5p was used as a control in this study. The average ± s.d. of two individual experiments are listed above as the data points. One-way analysis of variance (ANOVA) was performed to determine significant differences between the expression of miRNAs in SARS-CoV-2 infected and the uninfected groups. *p* value of 0.05 or less was considered statistically significant. '**' and 'ns' denotes p < 0.0012 and not significant, respectively.

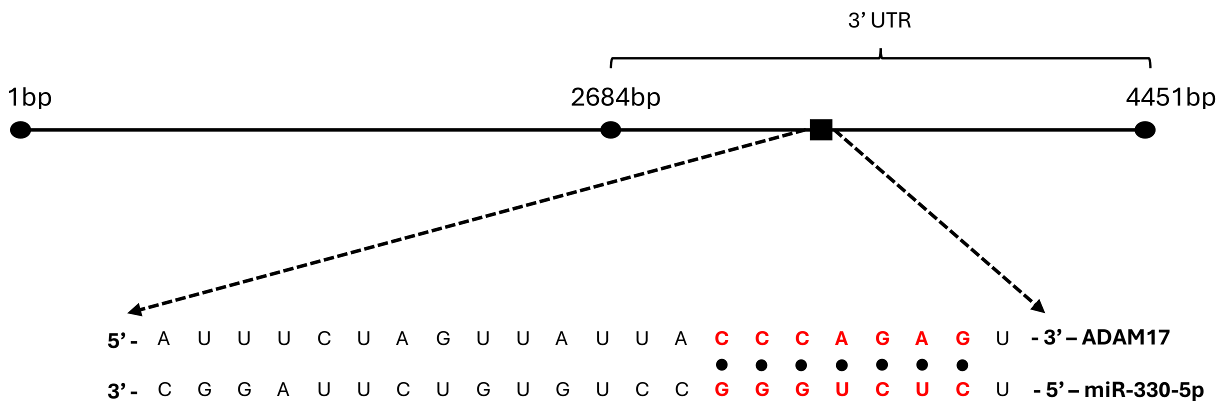

Supplementary Figure 3. A schematic showing the predicted putative binding sites (……bp) of miR-330-5p in the 3' UTR region of ADAM17 gene. The binding sites are highlighted in RED.

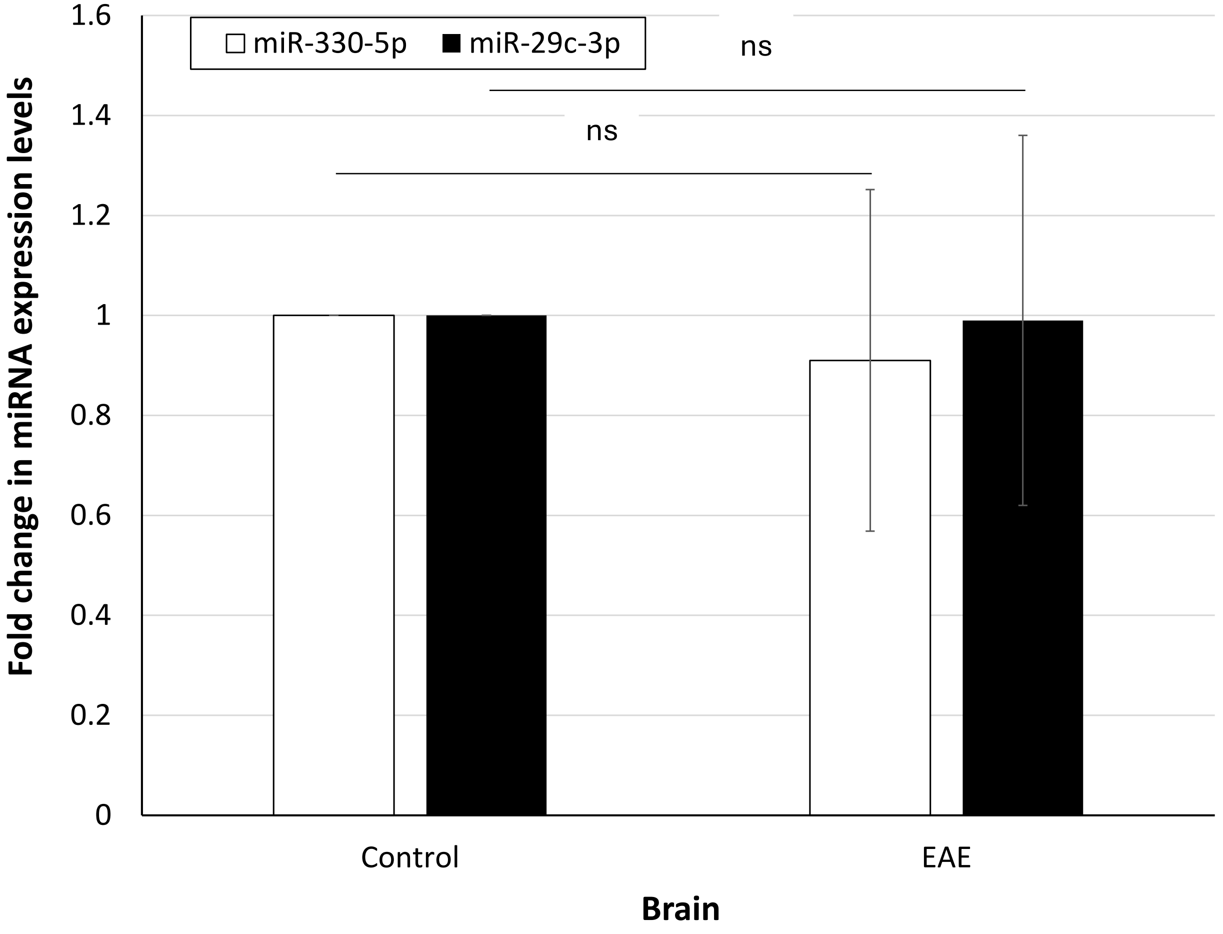

Supplementary Figure 4. Fold change in the miR-330-5p expression in EAE mice brain (*n = 6*) compared to control mice (*n = 6*) and that is considered to be 1-fold. Expression of miR-29c-3p was used as a control in this study. The average ± s.d. of two individual experiments are listed above as the data points. One-way analysis of variance (ANOVA) was performed to determine significant differences between the expression of miRNAs in EAE mice brain compared to control mice brains. *p* value of 0.05 or less was considered statistically significant. 'ns' denotes not significant.
